# Association analysis between agronomic traits and AFLP markers in a wide germplasm of proso millet (*Panicum miliaceum* L*.*) under normal and salinity stress conditions

**DOI:** 10.1186/s12870-020-02639-2

**Published:** 2020-09-15

**Authors:** Mehdi Yazdizadeh, Leila Fahmideh, Ghasem Mohammadi-Nejad, Mahmood Solouki, Babak Nakhoda

**Affiliations:** 1 Department of Plant Breeding and Biotechnology, Faculty of Agriculture, University of Zabol, Zabol, Sistan and Baluchestan province Iran; 2 Department of Agronomy and Plant Breeding, College of Agriculture, Shahid Bahonar University of Kerman, Kerman, 76169-133 Iran; 3 Department of Molecular Physiology, Agricultural Biotechnology Research Institute of Iran, Mahdasht Rd, Karaj, 31535-1897 Iran

**Keywords:** Amplified fragment length polymorphism, Genetic linkage, Population structure, MLM model, Marker stability

## Abstract

**Background:**

Proso millet is a highly nutritious cereal considered an essential component of processed foods. It is also recognized with high water-use efficiency as well as short growing seasons. This research was primarily aimed at investigating the genetic diversity among genotypes based on evaluating those important traits proposed in previous researches under both normal and salinity- stress conditions. Use of Amplified fragment length polymorphism (AFLP) molecular markers as well as evaluating the association between markers and the investigated traits under both conditions was also another purpose of this research.

**Results:**

According to the phenotypic correlation coefficients, the seed yield had the highest correlation with the forage and biological yields under both conditions. By disintegrating those traits investigated under normal and salinity-stress conditions into principal component analysis, it was found that the first four principal components justified more than 59.94 and 62.48% of the whole variance, respectively. The dendrogram obtained by cluster analysis displayed three groups of genotypes under both normal and salinity- stress conditions. Then, association analyses were conducted on 143 proso millet genotypes and 15 agronomic traits as well as 514 polymorphic AFLP markers (out of 866 created bands) generated by 11 primer combinations (out of the initial 20 primer combinations) EcoRI/MseI. The results obtained by mixed linear model (MLM) indicated that under normal conditions, the M14/E10–45 and M14/E10–60 markers had strong associations with seed yield. A similar trend was also observed for M14/E10–45 and M14/E11–44 markers in relation to forage yield. On the other hand, M14/E10–14, M14/E10–64 markers (for seed yield) and M14/E10–64 marker (for forage yield), had significant and stable association in all environments under salinity-stress conditions. Moreover, a number of markers showed considerable associations and stability under both normal and salinity stress conditions.

**Conclusions:**

According to the analysis of phenotypic data, the wide germplasm of Iranian proso millet has significant variation in terms of measured traits. It can be concluded that markers showing strong associations with traits under salinity-stress conditions are suitable candidates to be used in future marker-assisted selection (MAS) studies to improve salinity-resistance genotypes of *Panicum miliaceum* in arid and semiarid areas.

## Background

The genus proso millet (*Panicum miliaceum* L.), belongs to the Paniceae family in the Panicoideae subfamily. As an ancient grain crop, its historical reports date back to 10,000 years ago and has been the major cultivated grain crop in Europe since 2000 [1]. Today, it is produced in Eastern Europe, Russia, China, India, and North America [2–4]. Millet is one of those crops with the shortest growth periods (60–90 days) compared to other cereals. Moreover, its drought-resistance and hot spring forage plant makes it adaptable to challenging environmental conditions. It is also a low-maintenance and stress-resistant plant producing acceptable yield, making it appropriate for crop production in inhospitable climates [5–7]. Furthermore, it contains special alkaline proteins and a relatively balanced array of rare elements and vitamin precursors that exceed the levels in products such as wheat, rice and barley. Therefore, millet has remained an important component of the human diet [7–9].

Water-related impacts including climate change effects, namely water scarcity, increased agricultural land salinity, heightened intensity and longer drought periods as well as rising need for plant products led to further investigation into agriculture under saline conditions in countries like Iran [10–14]. Salinity stress causes a wide range of reactions in plants. Change in gene expression and cell metabolism, variation in plant growth rates and yields, reduction in the biomass production and efficiency of photosynthesis as well as altering leaf turgidity are among the main consequences of salinity stress [15].

Along with ever-increasing use of modified substances inside germplasm with the purpose of developing plants, the methods employed for classification and management of genetic diversity have received increasing attention. In this context, using statistical multi-variable approaches is considered an important strategy in germplasm classification, managing diversity among a large number of samples as well as evaluating the genetic associations in those investigated substances [16–19]. Cluster analysis, as one of multi-variable methods and initially employed to classify members based on their respective traits is used to mathematically organize individuals in a specific cluster. In this method, those members held in a cluster have the highest similarity or uniformity. This is while the highest level of difference or non-uniformity is observed between separate clusters. Therefore, those members inside a cluster would genetically be closer to each other while clusters with higher differences would have further distances in a diagram [20]. A cluster is assumed acceptable if i) a group of two or more genotypes has a within-cluster genetic distance of less than that of the overall mean ii) the genetic distance between two clusters is greater than that of within- cluster distance. Principal component analysis is one of the main characteristics of multi-variable approaches. This method, also used as a technique to reduce data into a limited number of non- associated variables, is employed to better understand the relationships among two or more traits. Furthermore, it can be employed to realize the differences among members and identify probable groups [21]. Identifying correlation among yields is an effective tool to determine valuable genotypes. High correlation and an acceptable heritability are essential criteria to select a trait for breeding programs [22]. To this end, practical statistics are appropriate approaches to determine the associations among genetic markers and phenotypic data via Genome-wide association study (GWAS). Therefore, such approaches increasingly facilitate use of genetic resources to improve yields [23].

Since phenotype is influenced by the environment, molecular methods such as DNA marker procedure are effective in molecular description of complicated traits [24]. It is noteworthy that DNA-based markers are the most appropriate methods to estimate genetic variation. Furthermore, markers which exhibit higher levels of diversity will have higher efficiency. The AFLP method not only needs background knowledge on the targeted genome but also has high reproducibility and sensitivity for finding polymorphism at various levels of the DNA sequence while provides valuable information in various loci of the targeted genome. Thus, the AFLP markers have been used in molecular and genetic studies in recent years [25, 26]. Kumar et al. [26] reported that AFLP is an indicator of genetic categorization, manufacturing of linkage maps, mapping of essential agronomic traits and devoting parentage.

Association analysis is extensively adopted to explore and identify relationships among molecular markers and agronomic traits based on linkage disequilibrium (LD) [27]. The accuracy of linkage analysis is influenced by many factors such as the magnitude of polymorphism between two parents, the population size, the distribution of chiasma in a genome and the time needed to produce artificial populations. Nevertheless, association analysis does not require pure populations. Besides, natural populations are used to find associations between markers and traits in this method. By establishing the recombination of these populations, the relationship between the markers and the traits would be accurate and reliable [28, 29].

Association analysis is performed using both general linear model (GLM) and mixed linear model (MLM) [30]. In GLM model, the marker is considered a constant variable and causes the first type to be incorrect. Therefore, a fake association is formed between the marker and trait. These types of errors are partly resolved using the Q matrix derived from the structure of the population. This matrix expresses the probable rate based on which each element can be attributed to the sub-structures. It also prevents incorrect associations between traits and markers which in turn greatly reduces type-1 error [26, 30, 31]. Meanwhile, both factors are used in MLM model by combining Q and K matrices to obtain more power compared to linear modeling. The K matrix expresses the kinship association of individuals in the population [32]. In order to evaluate the DNA polymorphism and genetic diversity among three domestic and nine wild proso millet biotypes, eight primer combinations and 39 polymorphic DNA fragments were identified [33, 34]. Le Thierry d’ Ennequin et al. [35] found that AFLP markers could be used to evaluate the genetic association between foxtail millet and green foxtail. Colosi and Shaal [36] also used the random amplified polymorphic DNA (RAPD) marker to evaluate the genetic diversity among 97 species including 69 wild proso millets, 26 crops and crop-like weeds as well as two hybrid genotypes. Rajput et al. [37] performed association analysis using 548 SSR markers. Their study led to the identification of 339 polymorphics in 8 proso millet genotypes. Ebrahimi et al. [38] performed association analysis on eight important traits and 341 polymorphic AFLP markers produced by 10 primer combinations (EcoRI/MseI) among 100 safflower genotypes.

Little investigation has been carried out on the association analysis of proso millet agronomic traits using a large number of markers. The association analysis of proso millet agronomic traits is performed using AFLP as a molecular marker. This study involves a significant number of ecotypes under both normal and salinity-stress conditions. Use of this approach might be useful to improve the efficiency of marker-assisted selection and other breeding projects. The targets of the present study are: (i) investigating the genetic diversity among genotypes based on evaluation of those important traits proposed in previous researches under normal and salinity- stress conditions; (ii) evaluation of population genetic structures to detect essential marker-trait associations of the genotypes; (iii) the effectiveness of AFLPs in recognizing the loci marker association with significant agronomic traits of Iranian species subjected to normal and salinity-stress conditions, separately; and (iv) determining the stability of respective loci markers with the desired agronomic traits corresponding to both conditions.

## Results

### Analysis of phenotypic data

Analysis of variance (ANOVA) was performed through the PROC ANOVA procedure of SAS 9.1. The results indicated that genotypes had significant differences in terms of all agronomic traits under both normal and salinity-stress conditions. Moreover, all traits except panicle length were considerably influenced by the environment as well as the genotype × environment interaction in all conditions (Additional file 1: Table S1). This implied a significant genetic variation in terms of traits and the possible selection and application of these traits in breeding programs.

Salinity stress reduced seed and forage yield by 20 and 12%, respectively. Regarding the investigated traits, proso millet cultivars had an extensive phenotypic variation. For example, the genotype G57 had the highest average percentage of seed germination while it had the lowest mean value for the number of tillers.

Under normal conditions, the highest quantities of seed and forage yields were determined 2.94 t/ha (G32) and 6.35 t/ha (G32), respectively. In addition, the lowest quantities were reported 1.56 t/ha (G141) and 3.35 t/ha (G141), respectively. Under salinity-stress conditions, the highest quantities of seed and forage yield were estimated 3.05 t/ha (G44) and 7 t/ha (G142) while the lowest quantities were reported 0.52 t/ha (G63) and 0.78 t/ha (G18), respectively (data not shown). The evaluated broad-sense heritability (h2) required to measure the traits of proso millet genotypes are given in Additional file 1: Table S1. The highest h2 value was observed in the 1000-seed weight (0.97 and 0.96 under normal and salinity-stress conditions, respectively) while the lowest h^2^ value was estimated for plant height (0.55 and 0.27 under normal and salinity-stress conditions, respectively) (Additional file 1: Table S1).

### Correlation coefficients analysis

According to the phenotypic correlation coefficients, the seed and forage yields had positive and significant correlations with all investigated traits except for the number of leaves, flag leaf length, flag leaf width and panicle length under normal conditions Moreover, the seed yield had the highest correlations with forage and biological yields as well as the seed germination percentage (Table 1). This is while the forage yield had the highest correlations with seed and biological yields as well as the seed germination percentage.

**Table 1:** Phenotypic correlation coefficients of investigated traits of proso millet genotypes under normal conditions

Code of traits	1	2	3	4	5	6	7	8	9	10	11	12	13	14	15
**1**	1														
**2**	0.25**	1													
**3**	0.13^ns^	0.14^ns^	1												
**4**	0.15^ns^	0.22**	0.14^ns^	1											
**5**	0.08^ns^	0.50^ns^	−0.13^ns^	0.15^ns^	1										
**6**	0.30**	0.20**	0.15^ns^	0.02^ns^	0.01^ns^	1									
**7**	0.05^ns^	0.04^ns^	0.07^ns^	0.08^ns^	0.36**	0.04^ns^	1								
**8**	0.71**	0.38**	0.13^ns^	0.12^ns^	0.05^ns^	0.31**	0.12^ns^	1							
**9**	0.29**	0.25**	0.01**	0.16*	0.12^ns^	0.22**	0.19*	0.26**	1						
**10**	0.31**	0.26**	−0.03^ns^	0.13^ns^	0.12**	0.34**	0.04^ns^	0.28**	0.30**	1					
**11**	0.11**	0.39**	0.13^ns^	0.12^ns^	0.05^ns^	0.31**	0.12^ns^	0.99**	0.26**	0.28**	1				
**12**	0.17*	0.05^ns^	−0.04^ns^	0.15^ns^	0.13^ns^	0.07^ns^	0.27**	0.23**	0.08^ns^	0.09^ns^	0.22**	1			
**13**	0.28**	0.33**	^ns^06.0	0.19**	0.13^ns^	0.20*	0.20*	0.30**	0.31**	0.28**	0.30**	0.23**	1		
**14**	−0.27**	− 0.21**	− 0.14^ns^	0.16^ns^	0.07^ns^	− 0.27^ns^	− 0.09^ns^	− 0.21*	− 0.05^ns^	− 0.04^ns^	− 0.23*	0.23**	−0.12^ns^	1	
**15**	0.72**	0.39**	0.13^ns^	0.12^ns^	0.05^ns^	0.31**	0.12^ns^	0.99**	0.26**	0.28**	0.99**	0.22**	0.30**	−0.20**	1

ns, *, **: not significant, significant at 0.05 and 0.01 level, respectively

Under salinity-stress conditions, the seed yield demonstrated positive and significant correlations with all traits except for flag leaf width and panicle length. Moreover, the seed yield had the highest correlations with biological and forage yields as well as the main panicle seeds weight. This is while the forage yield demonstrated the highest correlations with biological and seed yields as well as the panicle seeds weight under similar conditions. Besides, the forage yield had no significant correlations with flag leaf width, panicle length and harvest index (Table 2).

**Table 2:** Phenotypic correlation coefficients of investigated traits of proso millet genotypes under salinity stress conditions

Code of traits	1	2	3	4	5	6	7	8	9	10	11	12	13	14	15
**1**	1														
**2**	**340.	1													
**3**	*170.	^ns^110.	1												
**4**	*180.	**380.	^ns^050.	1											
**5**	^ns^060.	^ns^140.	^ns^130.-	^ns^120.	1										
**6**	**270.	**310.	**190.	**270.	^ns^060.	1									
**7**	^ns^0050.	^ns^0740.	^ns^130.-	ns030.	0.30**	**030.	1								
**8**	0.05**	**430.	*170.	**220.	^ns^060.	**580.	^ns^140.	1							
**9**	**240.	**470.	^ns^070.	*160.	^ns^140.	**320.	*190.	**460.	1						
**10**	**450.	**290.	^ns^030.	**020.	^ns^140.	**350.	^ns^020.	**490.	**370.	1					
**11**	**450.	**420.	*170.	**280.	^ns^060.	0.60**	^ns^140.	**850.	**360.	**430.	1				
**12**	**270.	**230.	^ns^040.	0.10^ns^	^ns^080.	^ns^070.	**210.	**650.	**240.	**290.	0.50**	1			
**13**	**0.28	**310.	^ns^070.	**29/	^ns^130.	**220.	**020.	0.50**	**240.	0.40**	**490.	**050.	1		
**14**	**190.	^ns^060.	^ns^030.-	^ns^080.-	^ns^060.-	^ns^070.	^ns^020.	**310.	^ns^090.	^ns^130.	^ns^180.-	**350.	^ns^110.	1	
**15**	**480.	**440.	*170.	**270.	^ns^060.	**610.	^ns^150.	0.93**	0.39**	0.46**	0.98**	0.57**	0.52**	0.05^ns^	1

ns, *, **: not significant, significant at 0.05 and 0.01 level, respectively

### Principal component analysis

By disintegrating those traits investigated under normal conditions into principal component analysis and taking into account the eigenvalues of larger than unity; it was found that the first four principal components justified more than 59.94% of the whole variance. Furthermore, the first, second, third and fourth components constituted 31.22, 11.91, 8.94 and 7.87% of the whole variance, respectively (Table 3). The seed germination, seed yield as well as forage and biological yields had the highest coefficients in the first principal component. Moreover, the first principal component demonstrated positive correlations with the above mentioned traits. Therefore, determining genotypes with the highest values of the first principal component could lead to the identification of those with the highest potential yields under normal conditions. The panicle length, flag leaf width, number of panicle branches, 1000-seed weight and harvest index had the highest coefficients in the second principal component (Table 4). Therefore, this principal component, having smaller share in variance compared to the first one, would have stronger correlations with growth and reproductive properties to produce seeds. The positive and significant correlations of these traits with the second principal component indicated that those genotypes with higher values of the second principal component would have greater growth and reproduction capability. The plant height, number of leaves, number of tillers, number of panicle branches and number of plants on the line and 1000-seed weight had the highest positive coefficients in the third principal component (Table 4). This is while the plant height, flag leaf length and harvest index justified the most variance of the forth-principal component. Therefore, those genotypes with the highest values of the third and fourth principal components would have more such traits (Table 5).

**Table 3:** The principal component values of proso millet genotypes under normal conditions

Number of PCA	Eigenvalue	Variability (%)	Cumulative %
**1**	4.68	31.22	31.22
**2**	1.79	11.91	43.13
**3**	1.34	8.94	52.07
**4**	1.18	7.87	59.94
**5**	0.98	6.54	66.49
**6**	0.93	6.22	72.71
**7**	0.78	5.23	77.94
**8**	0.71	4.70	82.64
**9**	0.68	4.55	87.19
**10**	0.60	3.97	91.16
**11**	0.53	3.50	94.66
**12**	0.49	3.26	97.92
**13**	0.30	2.07	99.99
**14**	0.01	0.01	100.00

**Table 4:** Members constituting the first five components based on investigated traits of proso millet genotypes under normal conditions

Code of traits	PCA 1	PCA 2	PCA 3	PCA 4	PCA 5
**1**	0.37	−0.09	−0.12	−0.01	−0.10
**2**	0.24	−0.02	0.29	0.23	0.00
**3**	0.08	−0.33	0.16	−0.13	0.73
**4**	0.10	0.31	0.00	0.57	− 0.09
**5**	0.07	0.41	0.11	−0.45	− 0.27
**6**	0.22	−0.11	0.34	−0.07	0.01
**7**	0.10	0.44	0.03	−0.47	0.08
**8**	0.43	−0.10	− 0.28	− 0.02	− 0.05
**9**	0.21	0.18	0.36	0.07	0.17
**10**	0.22	0.13	0.33	0.21	−0.19
**11**	0.43	−0.11	− 0.27	− 0.03	−0.06
**12**	0.13	0.39	−0.31	−0.04	0.41
**13**	0.23	0.23	0.29	0.08	0.23
**14**	−0.15	0.35	−0.33	0.33	0.26
**15**	0.43	−0.10	−0.27	− 0.02	−0.06

**Table 5:** Correlation of investigated traits with the first five components of proso millet genotypes under normal conditions

Code of traits	PCA 1	PCA 2	PCA 3	PCA 4	PCA 5
**1**	0.79	− 0.12	−0.14	− 0.02	−0.09
**2**	0.53	−0.03	0.34	0.25	0.00
**3**	0.18	−0.43	0.19	−0.15	0.72
**4**	0.22	0.42	0.00	0.62	−0.09
**5**	0.15	0.55	0.13	−0.49	−0.27
**6**	0.47	−0.15	0.40	−0.08	0.01
**7**	0.22	0.59	0.04	−0.52	0.08
**8**	0.92	−0.13	−0.32	− 0.02	−0.05
**9**	0.45	0.24	0.42	0.08	0.17
**10**	0.47	0.18	0.38	0.23	−0.19
**11**	0.92	−0.14	− 0.31	−0.03	− 0.06
**12**	0.29	0.53	−0.35	−0.04	0.41
**13**	0.50	0.31	0.34	0.09	0.23
**14**	−0.32	0.47	−0.38	0.36	0.26
**15**	0.92	−0.14	−0.31	− 0.03	−0.06

By disintegrating, those traits investigated under salinity-stress conditions into principal component analysis and taking into account the eigenvalues of larger than unity; it was found that the first four principal components justified more than 62.48% of the whole variance. Moreover, the first, second, third and fourth components constituted 35.79, 9.96, 9.50 and 7.23% of the whole variance, respectively. According to the results, seed germination, plant height, number of tillers, seed and forage yields along with 1000 seed-weight justified the highest variance of the first principal component (Table 6). As seen, these traits were more related with the capacity of plants to have higher yields under stress conditions. Therefore, this principal component could be described as yield potential. Moreover, a positive correlation was found between yield and this principal component (Table 7). Hence, selecting those genotypes with higher values of the first principal component would facilitate identifying those genotypes with higher yield potentials under stress conditions. Since leaf width, panicle length and number of tillers justified the highest portion of variance in the second principal component, this principal component could probably express the capacity of genotypes to assign extra photosynthesized materials to seeds production. Furthermore, flag leaf width and panicle length had the highest values of the third principal component while plant height, number of panicle branches as well as number of plants on the line constituted the highest values of the forth one. Therefore, these principal components would probably indicate the vegetative growth of genotypes. Regarding the highly positive correlations of the above mentioned traits with the second, third and fourth principal components, selecting those genotypes having the highest values of these principal components is highly recommended (Table 8).

**Table 6:** Members constituting the first five components based on investigated traits of proso millet genotypes under salinity stress conditions

Number of PCA	Eigenvalue	Variability (%)	Cumulative %
**1**	5.36	35.79	35.79
**2**	1.49	9.96	45.75
**3**	1.42	9.50	55.25
**4**	1.08	7.23	62.48
**5**	0.95	6.35	68.83
**6**	0.88	5.84	74.67
**7**	0.82	5.48	80.15
**8**	0.72	4.78	84.93
**9**	0.64	4.27	89.20
**10**	0.58	3.88	93.08
**11**	0.43	2.88	95.96
**12**	0.39	2.57	98.53
**13**	0.20	1.32	99.85
**14**	0.02	0.15	100.00

**Table 7:** Members constituting the first five components based on investigated traits of proso millet genotypes under salinity stress conditions

Code of traits	PCA 1	PCA 2	PCA 3	PCA 4	PCA 5
**1**	0.26	−0.14	0.06	0.27	0.04
**2**	0.25	0.06	−0.24	0.35	0.09
**3**	0.07	−0.44	−0.10	− 0.12	−0.21
**4**	0.16	0.10	−0.38	0.18	0.61
**5**	0.08	0.57	−0.18	0.06	−0.18
**6**	0.27	−0.17	−0.26	0.01	−0.34
**7**	0.09	0.58	0.06	−0.27	−0.27
**8**	0.40	−0.09	0.19	−0.09	−0.06
**9**	0.24	0.13	−0.14	0.35	−0.39
**10**	0.27	0.01	0.00	0.31	0.01
**11**	0.38	−0.11	−0.12	−0.35	− 0.03
**12**	0.27	0.12	0.45	−0.19	0.19
**13**	0.28	0.15	0.11	−0.14	0.39
**14**	0.09	−0.01	0.63	0.44	−0.08
**15**	0.40	−0.10	−0.02	− 0.27	−0.04

**Table 8:** Correlation of investigated traits with the first five components of proso millet genotypes under salinity stress conditions

Code of traits	PCA 1	PCA 2	PCA 3	PCA 4	PCA 5
**1**	0.59	− 0.18	0.07	0.28	0.03
**2**	0.59	0.07	−0.29	0.37	0.08
**3**	0.17	−0.54	−0.12	− 0.12	−0.20
**4**	0.38	0.12	−0.46	0.19	0.59
**5**	0.18	0.69	−0.22	0.06	−0.17
**6**	0.63	−0.21	−0.31	0.02	−0.33
**7**	0.21	0.70	0.07	−0.28	−0.26
**8**	0.92	−0.11	0.22	−0.09	−0.06
**9**	0.54	0.16	−0.16	0.37	−0.38
**10**	0.62	0.01	0.00	0.32	0.01
**11**	0.88	−0.13	−0.14	−0.36	− 0.02
**12**	0.63	0.15	0.54	−0.20	0.19
**13**	0.64	0.18	0.13	−0.15	0.38
**14**	0.20	−0.02	0.75	0.46	−0.08
**15**	0.92	−0.13	−0.02	− 0.28	−0.04

### Cluster analysis

**Fig. 1:**
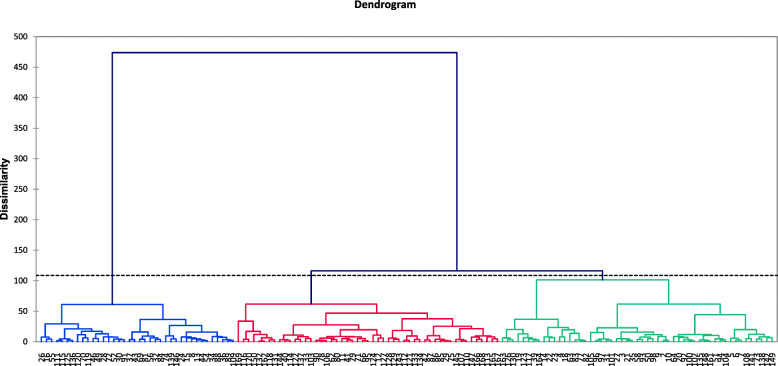
Cluster analysis-based dendrogram of investigated traits of proso millet genotypes under normal conditions

In this research, cluster analysis was used for grouping the lines. Under normal conditions, the tree diagram obtained by cluster analysis displayed three groups of genotypes. The first, second and third clusters were comprised of 54, 38 and 51 genotypes, respectively (Fig. 1) (Additional file 2: Table S2).

**Fig. 2:**
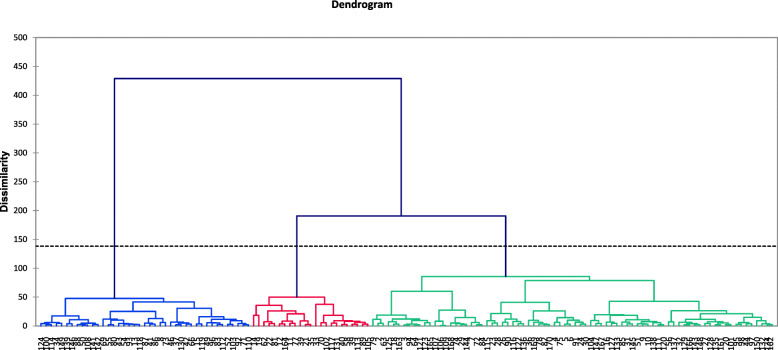
Cluster analysis-based dendrogram of investigated traits of proso millet genotypes under salinity stress conditions

Under salinity-stress conditions, the corresponding diagram displayed three groups of genotypes. The first, second and third clusters consisted of 79, 41 and 23 genotypes, respectively (Fig. 2) (Additional file 3: Table S3).

### Allele diversity

In total, 866 bands were created using 11 primer combinations, of which 514 bands were polymorphic. Since small alleles are usually used in LD assessment of pairs of loci [39], those alleles with a frequency of less than 0.05 were deleted before analysis. In this research, the Polymorphic Information Content index (PIC) ranged from 0.13 (M3/E10) to 1.21 (M14/E10) with a mean value of 0.6. Furthermore, the number of polymorphic bands for each primer combination varied from a minimum value of 12 (M3/E11) to a maximum value of 81 (M14/E10). On the other hand, the lowest polymorphic percentage (22.78%) was determined for corresponding M3/E10 primer combination. This is while the M14/E10 primer combination had the highest polymorphism of 97.59%. However, the average value of polymorphism was 58%. Moreover, the highest Shannon index (H) (2.20) was reported for M14/E10 primer combination while the lowest value (0.29) was determined for M3/E10 primer combination. Nonetheless, the average value of the Shannon index was determined 1.13. The primer combinations M3/E10 and M14/E10 also showed the lowest and highest marker index (MI), respectively (Table 9).

**Table 9:** Statistical variance of 11 AFLP primer combinations for the 143 proso millet genotypes

Primer combination	Total Bands	Polymorphic bands	Polymorphic percentage	PIC	Marker Index	Shannon index
M3/E10	79	18	22.78	0.13	2.34	0.29
M3/E11	51	12	23.52	0.25	3	0.43
M4/E10	81	22	27.16	0.16	3.52	0.35
M59/E10	83	37	44.57	0.48	17.76	0.90
M59/E36	83	67	80.72	0.97	64.99	1.78
M59/E11	82	61	74.39	0.65	39.65	1.28
M4/E11	83	74	89.15	1.06	78.44	1.93
M14/E11	82	43	52.43	0.65	27.95	1.18
M14/E10	83	81	97.59	1.21	98.01	2.20
M3/E36	81	50	61.72	0.57	28.50	1.13
M4/E36	78	49	62.82	0.47	23.03	0.96
Total	866	514	637	6.65	387.19	12.48
Mean	78.73	46.73	58	0.60	35.41	1.13

### Population structure

**Fig. 3:**
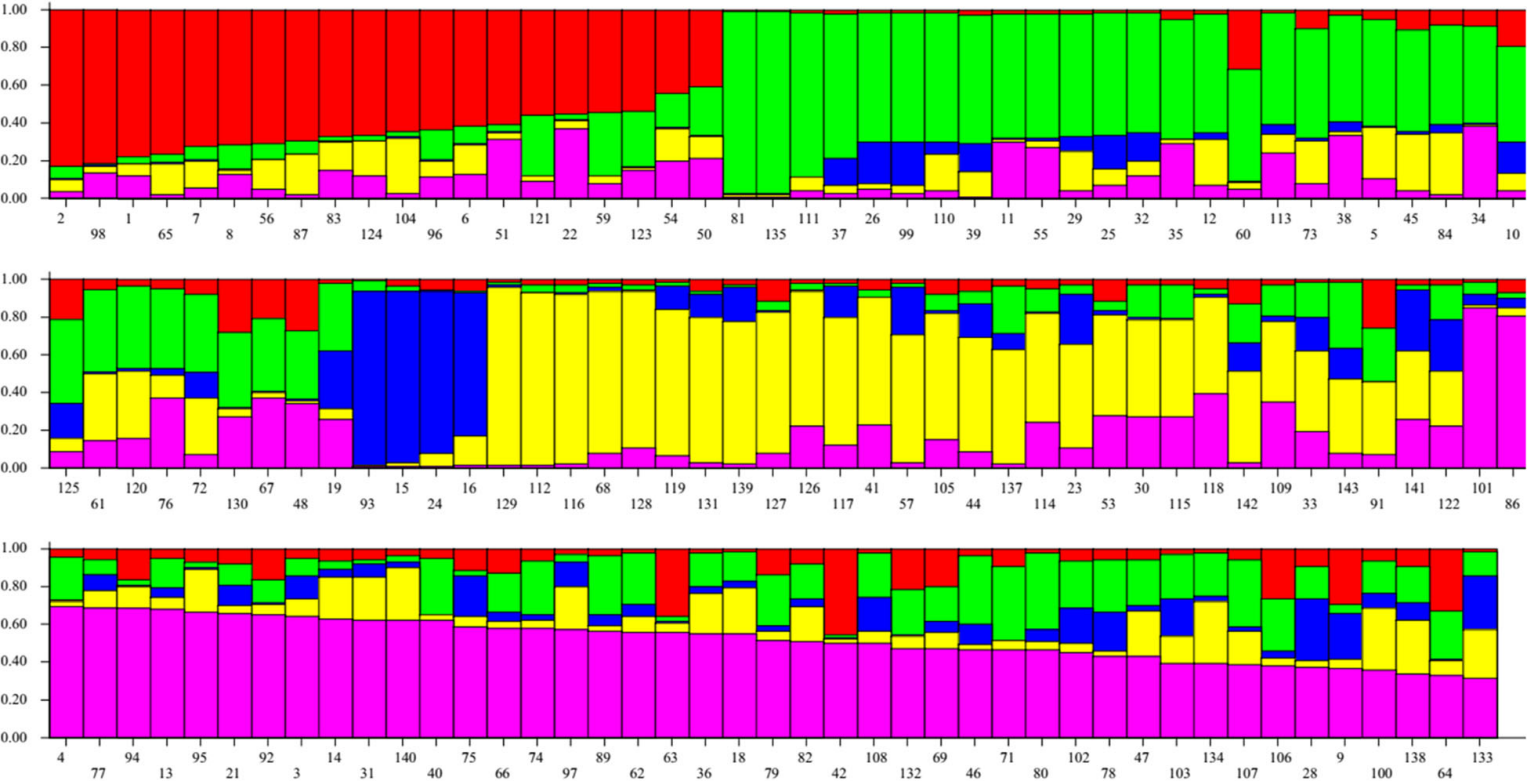
Biplot based on data obtained from 11 AFLP primer combinations using the structure software. Red, green, yellow, blue and violet colors indicate proso millet genotypes divided into five subpopulations

**Fig. 4:**
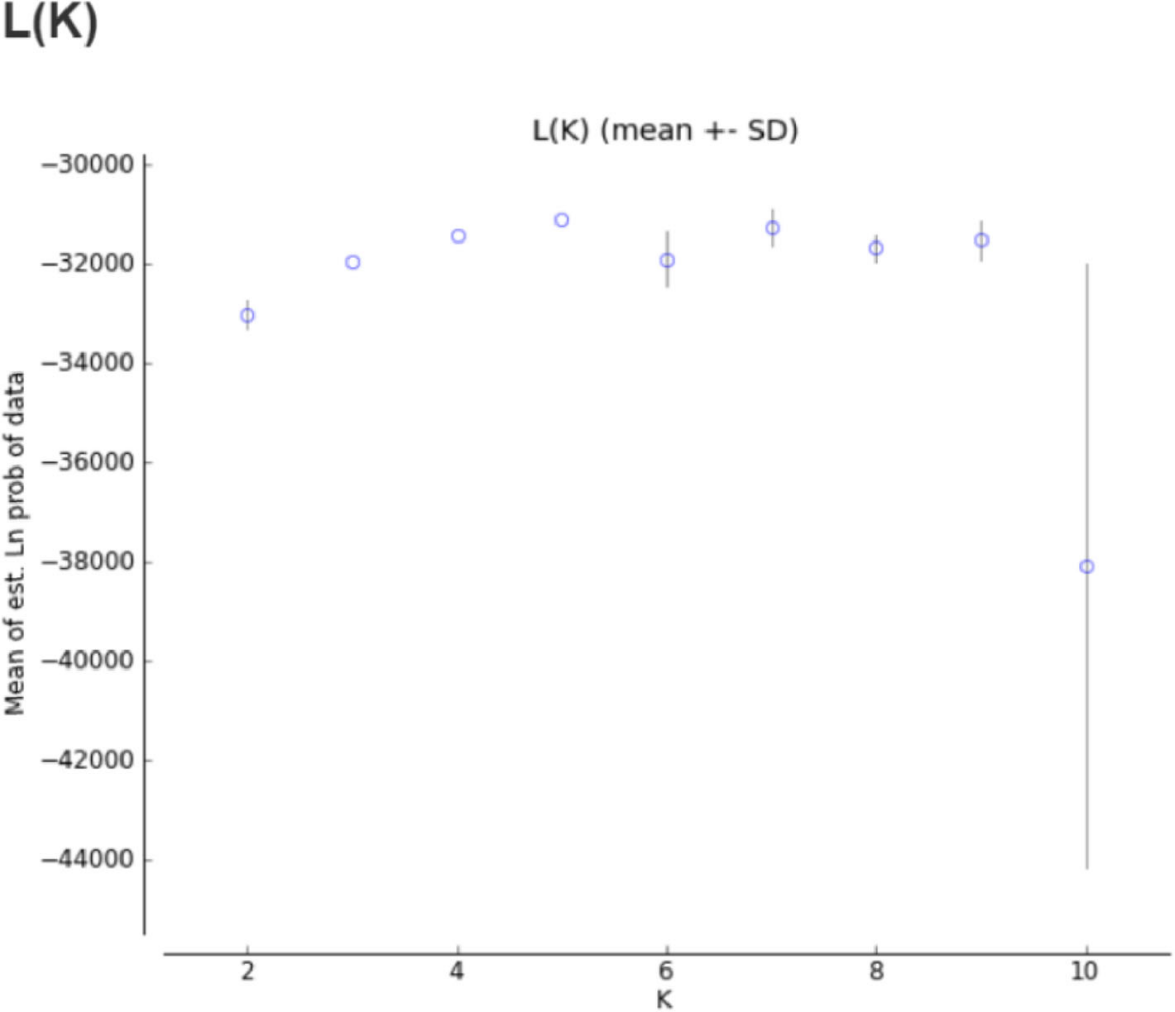
The graph (L(K), K) of Evanno’s method to determine the K values

**Fig. 5:**
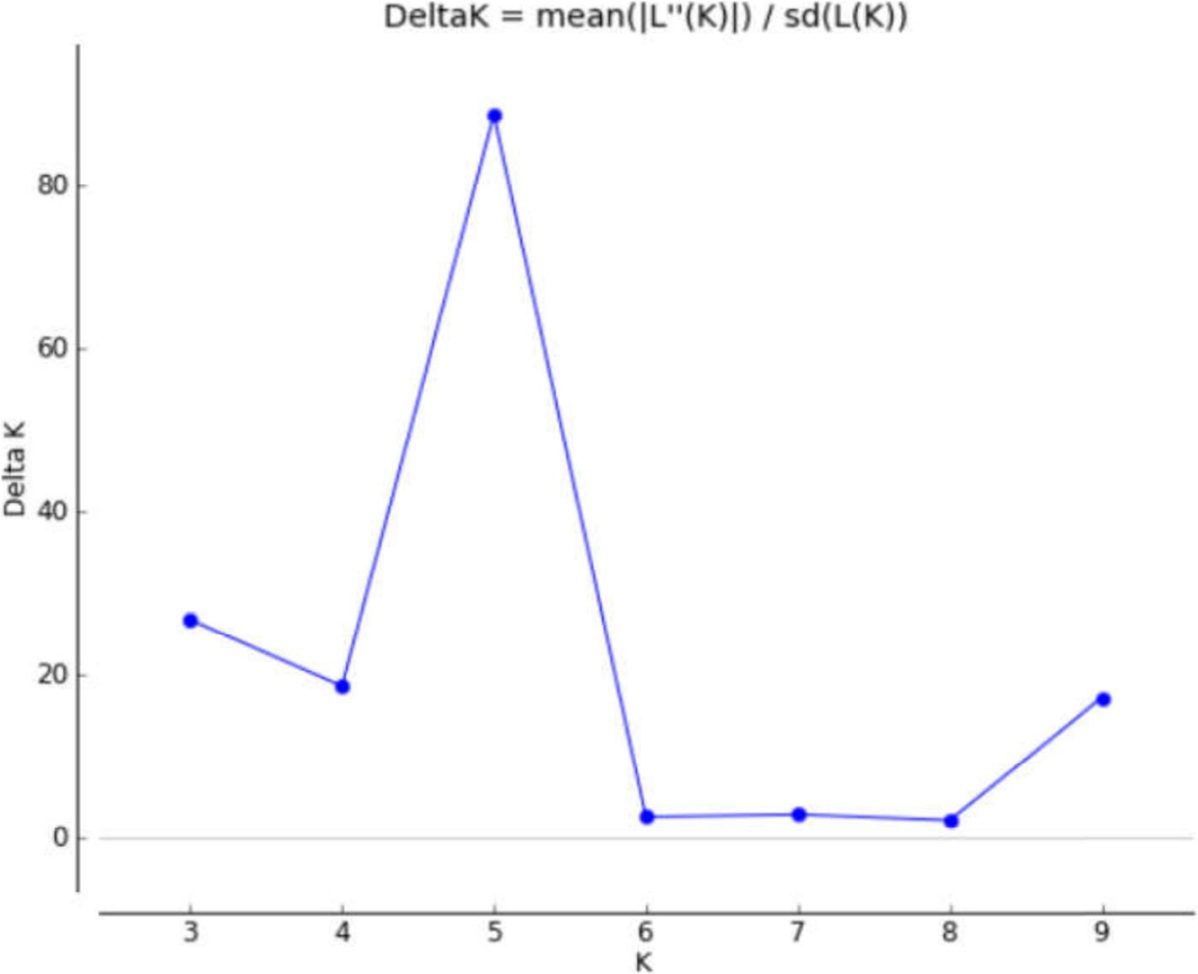
The graph (Delta K, K) of Evanno’s method to determine the K values

The number of clusters (K) present in proso millet (*Panicum miliaceum L*.) was determined by structure analysis. The value of ΔK was plotted versus the number of subpopulations (K) and structure analysis was subsequently performed based on the method adopted by Evanno et al. [40] The highest value of ΔK was observed at K = 5. According to the results obtained by HARVESTER STRUCTURE, the highest level of ΔK corresponded to K = five. Figures 3, 4 and 5 show the four stages used to determine the real value of K (Table 10).

**Table 10:** The results of Evanno’s method to determine the K values

K	Reps	Mean LnP(K)	Stdev LnP(K)	Ln’ (K)	Ln” (K)	ΔK
2	5	−32,995.26	279.05	–	–	–
3	5	− 3194.04	19.20	1048.22	513.46	26.73
4	5	−31,412.28	11.47	534.76	213.60	18.60
5	5	−31,091.12	12.64	32.16	1119.96	88.58
6	5	−31,889.92	548.95	− 798.80	1425.94	2.59
7	5	−31,262.78	358.87	627.14	1039.64	2.89
8	5	−31,675.28	262.19	−412.50	574.46	2.19
9	5	−31,513.32	392.33	161.96	6710.34	17.10
10	5	−38,061.70	608.50	6548.38	–	–

The yellow colored row represents K at maximum values of ΔK

It was observed that the contribution of the variance among and within the sub-populations were 7 and 93% of the total variance, respectively (Table 11). Moreover, the analogue stabilization index (PhiPT) values were significant, highlighting considerable genetic variations among subpopulations. On the other hand, the PhiPT values of each pair of subpopulations indicated significant differences among all subpopulations.

**Table 11:** Molecular variance (AMOVA) of association analysis of AFLP markers in proso millet genotypes by using the structure software

Source of variation	Df	MS	Est. Var	%	PhiPT
Among populations	4	153.08	3.76	7%	0.067**
Within populations	138	53.90	53.90	93%	
Total	142		57.67	100%	

**: Significant at 0.01 levels

### Association analysis

According to the association analysis performed by MLM model, the number of markers showing significant relationship with the average of the investigated traits was determined 67 and 65 under normal and salinity- stress conditions, respectively (Additional file 4: Table S4). However, kinship or affinity was not expected as an agent in GLM model. Moreover, the number of considerable markers increased to 93 and 99 under normal and salinity-stress conditions, respectively (data not shown). Based on the results obtained by MLM model, the determination coefficient (R^2^) ranged from about 3.28 to 5.45% under normal conditions and 3.33 to 5.21% under salinity-stress conditions. Furthermore, the values of R^2^ model ranged from 35.92 to 43.71% under normal conditions and 36.57 to 43.3% under salinity-stress conditions. However, the results obtained by GLM model revealed that the R^2^ varied from 5.22–9.37% to 5.2–9.97% under normal and salinity-stress conditions, respectively. Moreover, the values of R^2^ model varied from 5.81 to 12.3% under normal conditions and 5.55 to 14.28% under salinity-stress conditions (data not shown).

According to MLM model, the M14/E10–60, M14/E10–45, M4/E11–71, M4/E11–70 and M59/E36–1 markers with corresponding variations of 40.58, 40.47, 40.30, 40.15, and 40.09% demonstrated a significant relationship (*P* < 0.009) with seed yield under normal conditions. Nonetheless, the M14/E10–64, M14/E11–4 and M3/E36–2 markers with corresponding variations of 37.37, 36.94 and 36.57% revealed such relationship with seed yield under salinity-stress conditions (Additional file 4: Table S4).

Association analysis conducted on proso millet (*Panicum miliaceum* L*.)* accessions revealed that M14/E10–45, M14/E10–60, M4/E11–44, M4/E11–71, M59/E11–70 and M59/E11–75 markers with corresponding variations of 40.83, 40.52, 40.31, 40.18, 40.14, and 40.13% demonstrated a significant relationship (*P* < 0.008) with forage yield under normal conditions. However, the M14/E10–64, M3/E36–32 and M14/E10–78 markers with corresponding variations of 38.17%, 38.03 and 37.60% demonstrated a significant relationship (*P* < 0.008) with forage yield under salinity-stress conditions (Additional file 4: Table S4).

Five markers (M3/E10–3, M59/E36–3, M59/E11–47, M14/E10–69 and M14/E10–40) with corresponding variations of 40.99, 40.10, 40.03, 39.74 and 30.73% had strong relationship with the number of leaves per plant under normal conditions while seven markers (M4/E10–8, M59/E36–3, M59/E36–71, M3/E10–3, M59/E36–74, M14/E10–27 and M14/E10–40) with respective variations of 41.46, 41.31, 41.30, 41.25, 41.01,40.92 and 40.66% had similar relationship with the number of leaves per plant under salinity-stress conditions (Additional file 4: Table S4).

Moreover, four markers (M4/E11–44, M59/E36–60, M4/E36–4 and M59/E36–76) with corresponding variations of 41.06, 40.55, 40.46 and 40.18% revealed a considerable association (*P* < 0.006) with plant height under normal conditions. However, three markers (M59/E10–23, M59/E11–45 and M59/E36–14) with respective variations of 38.78, 38.75% and 38.15 revealed such an association under salinity-stress conditions, respectively (Additional file 4: Table S4).

The results also indicated that the M4/E36–67, M14/E10–67, M4/E11–78, M14/E10–44 and M59/E10–64 markers with corresponding variations of 39.20, 39.16, 38.85, 38.67, and 38.24% demonstrated a significant relationship (*P* < 0.009) with seed germination percentage under normal conditions. The M3/E10–2, M4/E11–1, M4/E36–2, M14/E11–44, M4/E36–45 and M4/E10–79 markers with corresponding variations of 41.16, 40.84, 40.65, 39.99, 39.98% and 39. 80% demonstrated a significant relationship with seed germination under salinity-stress conditions (Additional file 4: Table S4).

Furthermore, the M59/E36–39 marker was found to have association with flag leaf length under normal conditions while three markers including M59/E11–20, M59/E11–55, M59/E10–44, M59/E10–24 and M4/E11–11 had association with flag leaf length under salinity-stress conditions (Additional file 4: Table S4).

The M4/E11–44, M59/E36–37, M3/E36–17, M4/E11–40, M3/E11–13 and M4/E11–61 markers with respective variations of 37.39, 36.73, 36.10, 36.04, 35.92 and 35.92% demonstrated significant association (*P* < 0.009) with the number of panicle branches under normal conditions. Nonetheless, the M4/E36–45, M3/E10–71, M4/E11–76, M4/E11–83, M59/E11–45 and M3/E36–41 with corresponding variations of 38.71, 38.36, 38.31, 37.83, 37.47 and 37.41% were found to have similar association under salinity-stress conditions (Additional file 4: Table S4).

Furthermore, two markers including M59/E10–83 and M59/E11–82 with respective variations of 42.73 and 42.49% revealed significant relationship with flag leaf width under normal conditions while three markers including M4/E11–15, M4/E11–25 and M14/E10–2 with corresponding variations of 42.80, 41.83 and 41.43% indicated a considerable association with the same trait under salinity-stress conditions (Additional file 4: Table S4).

According to the results, the M59/E10–83, M14/E10–31 and M59/E36–48 markers with corresponding variations of 42.95, 41.55 and 41.07% demonstrated a significant relationship with the number of tillers under normal conditions. In contrast, three markers including M4/E11–25, M4/E11–15 and M59/E36–48 with respective variations of 41.57, 41.28 and 40.97% showed significant relationship with the number of tillers under salinity-stress conditions (Additional file 4: Table S4).

The M4/E10–25, M59/E11–15, M4/E10–48 and M59/E36–81 markers with respective variations of 40.60, 40.30, 40, 39.65 and 40.87% demonstrated strong association with panicle length under normal conditions. However, the M4/E10–8, M4/E10–11, M59/E11–18 and M59/E36–31 markers with corresponding variations of 40.37, 39.83, 39.73, 39.51 and 40.46% had significant relationship with this trait under salinity-stress conditions (Additional file 4: Table S4).

The results also indicated that the M4/E36–67, M4/E11–45, M59/E11–9 and M14/E10–19 markers with respective variations of 40.87, 40, 39.86 and 39.51% had a significant relationship (*P* < 0.008) with the number of plants on the line under normal conditions. However, four markers including M14/E11–67, M3/E36–41, M4/E11–31 and M59/E11–39 with corresponding variations of 40.46, 39.86, 39.01 and 38.96% were found to have similar association with the number of plants on the line under salinity-stress conditions (Additional file 4: Table S4).

It is noteworthy that the M59/E10–22, M59/E11–31, M4/E11–71, M3/E36–45 and M4/E11–61 markers with respective variations of 39.91, 39.51, 39.33, 39.32 and 38.82% had a significant relationship with the main panicle seed weight under normal conditions. Six markers including M59/E10–22, M3/E36–45, M4/E11–71, and M59/E11–31, M4/E11–61 and M4/E11–72 with corresponding variations of 39.36, 38.85, 38.75, 38.68, 38.62 and 38.46% had similar relationship with the main panicle seed weight under salinity-stress conditions (Additional file 4: Table S4).

The association analysis further indicated that the M3/E36–45, M14/E11–27, M14/E11–44 and M14/E10–69 markers with corresponding variations of 38.78, 38.30, 38.08 and 37.63% were associated with 1000-seed weight under normal conditions while five markers including M3/E36–41, M14/E11–44, M14/E11–27, M59/E11–54 and M14/E10–69 with respective variations of 39.64, 39.04, 38.50, 38.46 and 38% had similar relationship with 1000-seed weight under salinity-stress conditions (Additional file 4: Table S4).

Besides, the M4/E11–44, M4/E11–79, M3/E10–31, M4/E36–31, M14/E10–3, M3/E10–2 and M14/E11–23 markers with corresponding variations of 43.71, 43.28, 43, 42.98, 42.79, 42.51 and 42.44% were found to have strong association with the harvest index under normal conditions. Five markers including M14/E11–23, M3/E36–41, M4/E36–44, M4/E10–8 and M3/E11–13 with respective variations of 43.30, 43.01, 42.91, 42.87 and 42.73% revealed a strong association with the harvest index (*P* < 0.004) under salinity-stress conditions (Additional file 4: Table S4).

According to the results, under normal conditions, the M14/E10–45, M14/E10–60, M4/E11–44, M4/E11–71, M59/E11–70 and M59/E11–75 markers with corresponding variations of 40.88, 40.56, 40.32, 40.24, 40.18, and 40.15% demonstrated a significant relationship with the biological yield while two markers including M14/E10–64 and M3/E36–2 with respective variations of 37.72 and 37.24% showed similar association with the investigated trait (Additional file 4: Table S4).

To define stable relationships, association analysis was individually performed for each place using MLM model. The results showed that M14/E10–45 and M14/E10–60 markers (for seed yield), M14/E10–45 and M4/E11–44 markers (for forage yield), M14/E11–27 marker (for 1000-seed weight), M59/E36–37 marker (for the number of panicle branches), M59/E36–39 marker (for flag leaf length), M59/E10–22 marker (for main panicle seed weight) and M59/E10–83 marker (for the number of tillers) had a significant and stable association in all environments under normal conditions.

On the other hand, the M4/E11–45 marker (for seed germination percentage), M14/E10–14, M14/E10–64 markers (for seed yield), M14/E11–44 marker (for 1000-seed weight), M14/E10–64 marker (for forage yield), M59/E36–37 marker (for the number of panicle branches), M3/E36–45 marker (for main panicle seed weight) and M59/E36–48 marker (for the number of tillers) had significant and stable association in all environments under salinity-stress conditions.

## Discussion

The association analysis method has advantages over the Quantitative Trait Locus (QTL) method. The main advantages include bi-parental population performance, clear increment mapping and decreased investigation period as well as taking into account more alleles [41]. This method is sometimes used for proso millet (*Panicum miliaceum* L*.)* especially in dealing with different environmental conditions such as the salinity stress. It is employed to locate salinity-derived genes and use of various traits. The optimum application of this information would lead to improvements in the efficiency of MAS projects. In this context, developing knowledge could help protect the germplasm resources and the diversity of inherited traits. Moreover, it facilitates the determination of appropriate plants by markers with the purpose of breeding programs and other genetic researches [42].

According to the analysis of phenotypic data, genotypes varied considerably around the measured traits. This also indicates a significant genetic variation among the investigated genotypes. Based on the results, all traits were highly influenced by the environment and the genotype × environment interaction under both normal and salinity-stress conditions. Mehrani et al. [43] studied 10 proso millet genotypes in three locations to evaluate the correlations between seed yield and major agronomic traits. They found positive and significant correlations between seed yield and traits such as number of tillers, number of leaves and straw yield. Their observations were in agreement with the results of this research. In another investigation carried out on proso millet genotypes, Sing and Rao found [44] positive and significant correlations between seed yield and major agronomic traits such as straw weight, plant weight, panicle length and the number of tillers. In another research conducted on 14 morphological traits corresponding to 39 foxtail millet cultivars, Reddy and Larshmi [45] observed positive correlations between seed yield and the number of tillers, the number of fertil tillers, the biological yield as well as harvest index. As harvest index and biological yield had the highest effect on the seed yield, it was recommended to use these two specific traits as criteria to select superior cultivars. Since the results of this research are consistent with findings of previous investigations, those traits evaluated in various researches deserve more attention for breeding programs as well as selecting genotypes with the purpose of improving seed and forage yields.

Cluster analysis and disintegration into the principal components are effective tools to distinguish proso millet genotypes. According to this research, by disintegrating into the principal components, the first four main principal components could justify 59.94 and 62.48% of the whole variance of the investigated traits under normal and salinity-stress conditions, respectively. These results are in agreement with findings of [46]. They declared that the analysis of the principal principal components demonstrated that plant height, seed yield, number of tillers and 1000 seed- weight could be employed to distinguish the superior of proso millet genotypes.

Based on the cluster analysis, all genotypes were categorized into three groups. Compared to other groups, the second group of genotypes had superior seed and forage yields. Besides, the common genotypes held in the second group (G8, G13, G15, G34, G37, G43, G46, G49, G52, G54, G55, G69, G88, G119, G139, G146) had significant superiority in terms of seed and forage yields under both normal and salinity-stress conditions. Therefore, they could be used as superior genotypes of proso millet germplasm in future breeding studies. Similar observations were made in a research conducted on Taiwan-based foxtail millet in which three clusters were determined [47]. In another investigation carried out on foxtail millet, six major clusters were obtained [48]. However, this research led to the identification of three clusters. The difference observed in the number of clusters could be attributed to the different plant species as well as the number of investigated genotypes.

Therefore, it is essential to perform experimental association analysis in different places. The substantial percentage of polymorphism indicated that using AFLP combination in this research could be useful to find proso millet (*Panicum miliaceum* L*.)* genotypes. The findings of this study are in agreement with the results of other investigations conducted on millet [35, 49].

According to the allele diversity discussed in this investigation, the M14/E10 primer combination had the highest level of polymorphic percentage as well as high PIC, MI and Shannon index. Therefore, this marker can be regarded as the best combination for accessions of proso millet (*Panicum miliaceum* L*.)*.

The structure analysis demonstrated that accessions could be divided into five groups with various genetic structures. Moreover, the results showed that use of AFLP marker might be useful and efficient for population structure analysis which is in agreement with Kumar et al. [26]. According to their study, the AFLP could be used as an indicator for genetic categorization, manufacturing of linkage maps, mapping of agronomic traits and devoting parentage. Moreover, the results of association analysis revealed that the number of considerable markers decreased in the MLM compared to the GLM model. The combination of population structure and kinship in the MLM model would decrease fake affirmative associations. This indicated that the identification of some alleles in the GLM model might be due to the genotypic association with the corresponding traits.

These results are in agreement with the investigations conducted by Yu et al. [30] and Dadras et al. [50]. Furthermore, according to the results, the determination coefficient obtained by MLM model was significantly reduced compared to the one achieved by GLM model. Thus, AFLP markers used by MLM model might be good candidates for future studies. These results are in agreement with the findings of Achleitner et al. [51].

Although markers must be validated by examining their effectiveness on definitive goal-oriented phenotypes among absolute populations with diverse genetic backgrounds [52], the markers indicating the greatest effect on the traits would be the best candidates in future MAS studies. Association analysis using the MLM model demonstrated that the M14/E10–45 and M14/E10–60 markers had significant relationship with forage and seed yields under normal conditions. Furthermore, the M14/E10–64 marker had a significant and permanent relationship with seed and forage yields in all environments under salinity-stress conditions. If markers have substantial effects on traits, they can be useful in programs such as MAS under salinity-stress conditions. The variation range of phenotypic traits was calculated separately for each marker. The lower values indicate that these complicated traits might be controlled by other genes with smaller effects. Moreover, a low range of R^2^ value for each trait might be attributed to insufficient density of markers, insignificant quantitative effect of markers, scarce alleles and complicated allelic interactions [53, 54].

According to the MLM model, the M14/E10–45 and M14/E10–60 markers simultaneously had significant association with seed and forage as well as biological yields under normal and salinity-stress conditions. Shi et al. [55] suggested local QTLs for yield and other traits. It is quite natural that yield is defined based on the accumulative effect of different traits. The genes known to be effective show the effects of polytrophic at least on one trait [56]. Furthermore, the association of M4/E11–44 marker with seed germination percentage, seed and forage yields, the association of the M4/E11–61marker with the number of panicle branches and seed weight of main panicle as well as the significant and permanent relationship of the M14/E10–64 marker with seed and forage yields in all environments under salinity-stress conditions could be attributed to pleiotropic effects or the multiple linked genes in that region which influence some of those traits. Therefore, these markers can be highly useful for breeding these traits under salinity-stress conditions. Moreover, the M14/E10–27 and M14/E10–40 markers (for the number of leaves) and the M4/E10–8 and M59/E11–18 markers (for panicle length) had a significant and stable association in all environments under salinity-stress conditions.

## Conclusions

According to the analysis of phenotypic data, the wide germplasm of Iranian proso millet (143 studied genotypes) varied significantly in terms of measured traits. Moreover, the effects of environment as well as the genotype × environment interaction were significant under both normal and salinity stress conditions. These results indicated the existence of a considerable diversity among the germplasm, facilitating the selection and classification of genotypes especially salinity stress- resistance ones. Therefore, the investigation of association analysis of these germplasms in different environmental conditions will be important.

The results of the association analysis conducted on the investigated traits of proso millet demonstrated that most of the markers which control the traits had an acceptable level of polymorphism and diversity under normal and salinity-stress conditions. Furthermore, the primer combinations used in this study showed a high percentage of polymorphism and a high level of reliability in terms of PIC, MI and Shannon indices. Therefore, the marker compounds employed in this study can be considered a powerful tool to distinguish proso millet genotypes.

According to the results obtained by MLM model, a number of markers showed a considerable association and stability under both normal and salinity- stress conditions. Moreover, a number of markers had a significant relationship with several traits in all environments which might be due to the pleiotropic effects or strong association of several genes which affect a number of traits. Therefore, the introduced markers of this study showing significant relations with traits under salinity stress conditions could be suitable candidates to be used in future MAS studies to improve salinity-resistance genotypes of *Panicum miliaceum* in arid and semiarid areas.

## Methods

### Plant materials

This research is a part of Iran’s comprehensive research program running at the Iranian center of excellence for drought-resistance crops at the University of Kerman. The 143 proso millet (*Panicum miliaceum* L*.*) genotypes (Additional file 5: Table S5) were supplied by the Iranian center of excellence for drought-resistance crops at the University of Kerman. All studied genotypes collected by the center of excellence were selected from various regions of Iran with a long history of millet cultivation. For sampling, all necessary measures were taken according to the recommendations provided by Gene Bank Guidelines. All genotypes were locally-cultivated and no wild types were used in this research. The salinity-stress related traits of Iranian proso millet are wider than those of international millet genotypes. The field used for the experiments is located in the Iranian center of excellence for drought and salinity-resistance crops at longitude 56°54΄ E and latitude 30°20΄ N. It is situated 1755 m above the sea level. The soil is clayey loam.

### Planting and performing salt stress treatment

The varieties were cultivated in two locations at the research institute of salinity stress (Shahid-Bahonar University of Kerman and Ekhtiyarabad fields) under two conditions (normal and saline) over a 2 year- long period (2017 and 2018). Experimental design was performed in randomized complete block with 3 replications. Irrigation treatment was conducted under (i) non-stress and (ii) salinity- stress conditions from the beginning of plant growth to the end of seed filling. The field preparation operations such as plowing, weeding, fragmentation, fertilization and irrigation were regularly conducted at appropriate time intervals over the whole period of the experiments. The soil pH was determined 7.41 and 8.1 in Kerman and Ekhtiarabad fields, respectively. Furthermore, the electrical conductivity of irrigation waters used in Kerman and Ekhtiarabad fields were estimated 2.5 and 8.7 dS/m, respectively.

### Genomic DNA extraction

The genomic DNA was extracted from pristine leaves based on the Cetyltrimethyl ammonium bromide (CTAB) Doyle and Doyle [57] method. DNA quantity control was performed using DNA spectrophotometer spectrum and agarose electrophoresis. Moreover, amplified fragment length polymorphism was conducted according to Vos et al. [58]. The amplification process was carried out by applying 11 superlative instructive EcoRI/MseI primer combinations (Table 12). Initially, five hundred ng of each DNA sample was digested with ten units of MseI (5 U) and EcoRI (5 U) enzymes. Then, these samples were treated with EcoRI and then incubated at 37 °C and 65 °C, each for 16 h, respectively. Besides, they were exposed to EcoRI and MseI enzymes in a thermocycler at 65 °C for 16 h. Ligated DNA was diluted with water at 1:8 ratios and the pre-amplification process was conducted using primers and selective nucleotides. The reaction was completed in a 25 μl volume. The products of the pre-amplification stage were treated with primers as well as three selective nucleotides. The final propagation stage was conducted by 13 cycles of 30 s at 94C, 30 s at 65C as touchdown with 0.7C lowering for each cycle, and 1 min at 72C. The PCR continued by another 20 cycles of 30 s at 94C, 30 s at 56C and 1 min at 72C, and one final cycle of extension at 72C for 10 min. In order to analyze the results of the PCR selective stage based on the 0 M700 method, the QIAxcel device and kit’s High Resolution (QIAGEN, Hilden, Germany) were employed. The QX adjustment marker (15 bp/1 kp) was applied in this process.

**Table 12:** Primer combinations used in the analysis of amplified fragment length polymorphism (AFLP) of proso millet genotypes

Code of primer combination	Code Sequence for MseI	Code Sequence for EcoRI
M14/E11	MseI Selective Primer+CTG	EcoRI Selective primer+AGC
M59/E36	MseI Selective Primer+CTA	EcoRI Selective primer+AGC
M4/E10	MseI Selective Primer+CTT	EcoRI Selective primer+AGC
M3/E11	MseI Selective Primer+CAA	EcoRI Selective primer+AGC
M4/E11	MseI Selective Primer+CTT	EcoRI Selective primer+AGC
M59/E11	MseI Selective Primer+CTA	EcoRI Selective primer+AGC
M59/E10	MseI Selective Primer+CTA	EcoRI Selective primer+AGC
M4/E36	MseI Selective Primer+CTT	EcoRI Selective primer+AGC
M3/E36	MseI Selective Primer+CAA	EcoRI Selective primer+AGC
M14/E36	MseI Selective Primer+CTT	EcoRI Selective primer+AGC
M3/E10	MseI Selective Primer+CAA	EcoRI Selective primer+AGC

### Phenotyping evaluation

The traits evaluated in this investigation included seed germination (%), plant height (cm), the number of leaves per plant, flag leaf length (cm), flag leaf width (cm), the number of tillers, panicle length (cm), main panicle seed weight (g/m^2^), the number of panicle branches, the number of plants on the line, 1000-seed weight, harvest index, forage yield (t/ha), biological yield (t/ha) and seed yield (t/ha). The last three traits including forage yield, biological yield and seed yield were initially measured based on g/m^2^ and then converted to ton per hectare (t/ha) (Table 13).

**Table 13:** Names and code of investigated traits in the 143 proso millet genotypes.

Trait	Code of traits	Unit
Seed germination	1	%
Plant height	2	cm
Number of leaves per plant	3	–
Flag leaf length	4	cm
Flag leaf width	5	cm
Number of tillers	6	–
Panicle length	7	cm
Main panicle seed weight	8	g/m^2^
Number of panicle branches	9	–
Number of plants on the line	10	–
Seed yield	11	t/ha
1000-seed weight	12	g/m^2^
Forage yield	13	t/ha
Harvest index, forage yield	14	%
Biological yield	15	t/ha

### Statistical analyses

#### Phenotypic data analysis

Analysis of variance (ANOVA) was conducted based on complete block design (RCBD). Since the data obtained via a randomized complete block (RCBD) design are comparable to the lattice (data not presented), the observed data were standardized for each trait and ANOVA was carried out deep-seated on RCBD by SAS software v. 9.1 [59]. Broad-sense heritability of essential agronomic traits related to salt tolerance for each experiment was estimated according to Nyquist [60]:

$$ {H}^2={\sigma^2}_g/\left({\sigma^2}_g+{\sigma^2}_{ge}/e+{\sigma^2}_{\varepsilon }/ re\right) $$

Where *σ*^2^_*g*_, *σ*^2^_*ge*_ and *σ*^2^_*ε*_ denote the genetic variance, genetic × environment interactive variance and the remaining error variance, respectively. Moreover, *e* and *r* are the number of environments and replicates per environment, respectively.

#### Correlation coefficients analysis

The phenotypic correlation coefficients are used to evaluate the relationships among yield and its members as well as those among members of a specific yield. In this section, the pair-wise phenotypic correlation for all traits were calculated using SAS software v.9.1. Then, their significance was tested.

#### Principal component analysis

Regarding diversity among the investigated genotypes, principal component analysis was used to determine the effect of each trait as well as the overall classification of genotypes. Besides, in order to better understand the genotypes’ behavior and having a more effective selection and determining the effectiveness of each trait under normal and salinity-stress conditions, this process was conducted separately using SAS software v.9.1.

#### Cluster analysis

For grouping the lines, cluster analysis was conducted by Ward’s method based on Cofenticcoefficient. Moreover, the squared Euclidean distance was employed as similarity index. The SAS software v.9.1 was used in this analysis.

#### Molecular data analysis

For each primer combination, expositive statistical analysis was initially performed using GenAlEx software v. 6.5b3 for each primer combination [61]. The marker index (MI) which indicates marker’s efficiency, [62], Shannon’s index (H), [63], and polymorphic information content (PIC) [64] were evaluated. However, the Shannon’s index (H) is among the most popular techniques used to evaluate the genetic diversity. These indicators were determined based on the following relations:

$$ PIC=1-{p}^2-{q}^2 $$

$$ MI= PIC\times number\ of\ polymorphic\ loci $$

$$ H=-1\times \left(p\times \mathit{\ln}\ (p)+q\times \mathit{\ln}(q)\right) $$

The symbols *p* and *q* are the frequency of prevailing and unvalued alleles, respectively.

Analysis of molecular variance (AMOVA), as a method to calculate F-statistics among and within subpopulations, was performed by GenAlEx software v. 6.5b3 [31, 65]. The PhiPT statistics (analogy of FST, fixation index) was employed to measure the genetic difference among subpopulations:

$$ PhiPT= AP/ WP+ AP $$

The symbols *AP* and *WP* denote the approximate variance among and within populations, respectively.

#### Population structure

Population structure was determined using STRUCTURE software v. 2.3.4 and performed with 10 replicates for each simulation from K = 2 to 10 followed by 100,000 Markov chain Monte Carlo (MCMC) iterations. Furthermore, the blend model and correlated allele frequencies were selected for this analysis. The optimum *K* was determined based on ΔK calculated by the following equation:

$$ \Delta  K=m\left|L"(K)\right|/\mathrm{s}\left[\mathrm{L}\ \left(\mathrm{K}\right)\right] $$

The web page of STRUCTURE HARVESTER processing uses an optimum estimate of *K* value representing the maximum value of ΔK [40, 66].

#### Association analysis

TASSEL software v. 4.2.1 [32] was employed to find considerable associations among the population-level allele frequencies and morphological traits. The association analysis was performed using both GLM and MLM models [30]. To this end, the GLM model and the most stringent MLM model were applied. The *P* matrix estimated in both normal and salinity-stress experiment was used for significant associations. Moreover, the *Q* matrix was calculated based on the structural analysis (at highest ΔK) and used as a variable to modify the population structure in both models. Also, TASSEL software v. 4.2.1 was used to find the kinship matrix (K-matrix) based on the effects of markers as well as the phenotype of traits [32].

## Supplementary information

**Additional file 1: Table S1.** Combined ANOVA of the 143 proso millet genotypes under two conditions (normal and salt stress)) with 3 replications in 2 environments.

**Additional file 2: Table S2.** Code of genotypes categorized into three main clusters resulting from cluster analysis under normal conditions.

**Additional file 3: Table S3.** Code of genotypes categorized into three main clusters resulting from cluster analysis under salinity stress conditions.

**Additional file 4: Table S4.** Association analysis of 143 proso millet genotypes under normal and salt stress conditions based on the MLM model**.**

**Additional file 5: Table S5.** Geographical location and code of the collected proso millet (*Panicum miliaceum* L.) genotypes.

## Data Availability

The dataset generated and analyzed during the study are included in this published article and its supplementary information files, or are available from the corresponding authors on reasonable request.

## Abbreviations

AFLP: Amplified fragment length polymorphism
MLM: Mixed linear model
GWAS: Genome-wide association study
GLM: General Linear Model
MAS: Marker-assisted selection
LD: Linkage disequilibrium
RAPD: Random amplified polymorphic DNA
PIC: Polymorphic Information Content index
H: Shannon index
MI: Marker index
PhiPT: Analogue stabilization index
QTL: Quantitative Trait Locus
CTAB: Cetyltrimethyl ammonium bromide
