# Supplementary material for: Association analysis between agronomic traits and AFLP markers in a wide germplasm of proso millet (Panicum miliaceum L.) under normal and salinity stress conditions
Source: BMC Plant Biol. 2020 Sep 15;20:427. doi: 10.1186/s12870-020-02639-2 (PMC7493190; doi:10.1186/s12870-020-02639-2)
Supplement: Supplementary file 1 — Additional file 1: Table S1. Combined ANOVA of the 143 proso millet genotypes under two conditions (normal and salt stress)) with 3 replications in 2 environments. [file 12870_2020_2639_MOESM1_ESM.docx]

| **Additional file 1: Table S1.** Combined ANOVA of the 143 proso millet genotypes under two conditions (normal and salt stress)) with 3 replications in 2 environments. | | | | | | |
| --- | --- | --- | --- | --- | --- | --- |
| **Source of variation** | **Seed germination percentage** | | **Plant height** | | **Number of leaves per plant** | |
| **Condition** | **Normal** | **Stress** | **Normal** | **Stress** | **Normal** | **Stress** |
| **Environment (E)** | 154942.77** | 184594.67** | 44383.73** | 53497.23** | 9889.94** | 6208.46** |
| **Replication/ E** | 211.39 | 1194.84 | 658.96 | 531.81 | 102.60 | 547.40 |
| **Genotype (G)** | 906.79** | 866.96** | 361.96** | 232.80** | 538.87** | 533.47** |
| **G × E** | 369.88** | 392.73** | 76.18** | 141.52** | 40.54** | 37.41** |
| **Error** | 6.52 | 16.29 | 39.61 | 56.96 | 1.38 | 4.89 |
| **Heritability** | 0.45 | 0.42 | 0.55 | 0.27 | 0.81 | 0.80 |
|  | | | | | | |
| **Source of variation** | **Flag leaf length** | | **Flag leaf width** | | **Number of tillers** | |
| **Condition** | **Normal** | **Stress** | **Normal** | **Stress** | **Normal** | **Stress** |
| **Environment (E)** | 1631.10** | 685.65** | 5.34** | 8.39** | 55.38** | 56.12** |
| **Replication/ E** | 78.44 | 534.17 | 0.24 | 0.24 | 19.10 | 19.28 |
| **Genotype (G)** | 62.087** | 61.57** | 0.14** | 0.16 ** | 8.47** | 8.69** |
| **G × E** | 26.42** | 26.36** | 0.003** | 0.003 ** | 1.40** | 1.70** |
| **Error** | 0.91 | 3.87 | 0.0003 | 0.0003 | 0.30 | 0.35 |
| **Heritability** | 0.43 | 0.41 | 0.93 | 0.93 | 0.64 | 0.61 |
|  | | | | | | |
| **Source of variation** | **Panicle length** | | **Seed yield** | | **Number of panicle branches** | |
| **Condition** | **Normal** | **Stress** | **Normal** | **Stress** | **Normal** | **Stress** |
| **Environment (E)** | 494.41** | 2500.98** | 36.44** | 51.07** | 56.41** | 56.44** |
| **Replication/ E** | 54.28 | 55.57 | 0.05 | 0.17 | 4.38 | 4.42 |
| **Genotype (G)** | 62.72** | 62.66** | 1.45** | 1.44** | 5.26** | 5.28** |
| **G × E** | 0.07^ns^ | 0.02^ns^ | 0.53** | 0.54** | 0.90** | 0.94** |
| **Error** | 21.37 | 21.61 | 0.05 | 0.003 | 0.29 | 0.33 |
| **Heritability** | 0.66 | 0.65 | 0.47 | 0.46 | 0.62 | 0.61 |
|  | | | | | | |
| **Source of variation** | **Number of plant on the line** | | **Forage yield** | | **Main panicle seed weight** | |
| **Condition** | **Normal** | **Stress** | **Normal** | **Stress** | **Normal** | **Stress** |
| **Environment (E)** | 1921.51** | 2232.46** | 53.28** | 85.36** | 26.22** | 28.60** |
| **Replication/ E** | 23.98 | 22.65 | 0.44 | 0.83 | 0.20 | 0.40 |
| **Genotype (G)** | 27.86** | 28.09** | 5.74** | 5.68** | 2.72** | 2.75** |
| **G × E** | 5.98** | 4.28** | 2.53** | 2.59** | 0.54** | 0.55** |
| **Error** | 0.49 | 0.51 | 0.01 | 0.01 | 0.002 | 0.001 |
| **Heritability** | 0.60 | 0.67 | 0.43 | 0.41 | 0.63 | 0.63 |
|  | | | | | | |
| **Source of variation** | **1000-seed weight seeds** | | **Harvest index** | | **Biological yield** | |
| **Condition** | **Normal** | **Stress** | **Normal** | **Stress** | **Normal** | **Stress** |
| **Environment (E)** | 2.85** | 3.13** | 3654.77** | 4199.77** | 177.86** | 268.49** |
| **Replication/ E** | 0.071 | 0.27 | 47.08 | 167.92 | 0.69 | 1.09 |
| **Genotype (G)** | 0.53** | 0.54** | 144.78** | 141.75** | 12.50** | 12.38** |
| **G × E** | 0.004** | 0.005** | 143.97** | 141.26** | 4.90** | 5.02** |
| **Error** | 0.0012 | 0.0007 | 0.85 | 0.92 | 0.02 | 0.03 |
| **Heritability** | 0.97 | 0.96 | 0.25 | 0.24 | 0.46 | 0.45 |
| ns, *, **: not significant, significant at 0.05 and 0.01 level, respectively. | | | | | | |
