# Supplementary material for: Association analysis between agronomic traits and AFLP markers in a wide germplasm of proso millet (Panicum miliaceum L.) under normal and salinity stress conditions
Source: BMC Plant Biol. 2020 Sep 15;20:427. doi: 10.1186/s12870-020-02639-2 (PMC7493190; doi:10.1186/s12870-020-02639-2)
Supplement: Supplementary file 2 — Additional file 2: Table S2. Code of genotypes categorized into three main clusters resulting from cluster analysis under normal conditions. [file 12870_2020_2639_MOESM2_ESM.docx]

**Additional file 2: Table S2.** Code of genotypes categorized into three main clusters resulting from cluster analysis under normal conditions.

| **Cluster 1** | **Cluster 2** | **Cluster 3** |
| --- | --- | --- |
| G1 | G8 | G11 |
| G2 | G13 | G12 |
| G3 | G15 | G29 |
| G4 | G16 | G33 |
| G5 | G24 | G40 |
| G6 | G26 | G41 |
| G7 | G28 | G42 |
| G9 | G30 | G62 |
| G10 | G32 | G66 |
| G14 | G34 | G71 |
| G18 | G36 | G75 |
| G19 | G37 | G78 |
| G20 | G43 | G79 |
| G21 | G44 | G80 |
| G22 | G46 | G85 |
| G23 | G49 | G86 |
| G27 | G52 | G87 |
| G31 | G54 | G90 |
| G35 | G55 | G95 |
| G39 | G56 | G103 |
| G50 | G69 | G106 |
| G51 | G70 | G107 |
| G58 | G72 | G110 |
| G63 | G74 | G112 |
| G64 | G81 | G113 |
| G73 | G84 | G114 |
| G77 | G88 | G116 |
| G82 | G89 | G118 |
| G83 | G91 | G121 |
| G92 | G109 | G122 |
| G93 | G111 | G124 |
| G94 | G115 | G127 |
| G96 | G119 | G128 |
| G97 | G120 | G129 |
| G98 | G125 | G131 |
| G100 | G136 | G132 |
| G101 | G139 | G133 |
| G102 | G146 | G134 |
| G104 |  | G137 |
| G105 |  | G143 |
| G108 |  | G144 |
| G117 |  | G145 |
| G123 |  | G147 |
| G126 |  | G150 |
| G130 |  | G162 |
| G135 |  | G165 |
| G138 |  | G166 |
| G141 |  | G167 |
| G142 |  | G168 |
| G148 |  | G169 |
| G149 |  | G170 |
| G161 |  |  |
| G163 |  |  |
| G164 |  |  |
