# Supplementary material for: Association analysis between agronomic traits and AFLP markers in a wide germplasm of proso millet (Panicum miliaceum L.) under normal and salinity stress conditions
Source: BMC Plant Biol. 2020 Sep 15;20:427. doi: 10.1186/s12870-020-02639-2 (PMC7493190; doi:10.1186/s12870-020-02639-2)
Supplement: Supplementary file 3 — Additional file 3: Table S3. Code of genotypes categorized into three main clusters resulting from cluster analysis under salinity stress conditions. [file 12870_2020_2639_MOESM3_ESM.docx]

**Additional file 3: Table S3.** Code of genotypes categorized into three main clusters resulting from cluster analysis under salinity stress conditions.

| **Cluster 1** | **Cluster 2** | **Cluster 3** |
| --- | --- | --- |
| G1 | G8 | G11 |
| G2 | G13 | G12 |
| G3 | G15 | G14 |
| G4 | G34 | G19 |
| G5 | G37 | G21 |
| G6 | G41 | G22 |
| G7 | G42 | G33 |
| G9 | G43 | G35 |
| G10 | G46 | G36 |
| G16 | G49 | G39 |
| G18 | G50 | G40 |
| G20 | G52 | G58 |
| G23 | G54 | G62 |
| G24 | G55 | G70 |
| G26 | G66 | G77 |
| G27 | G69 | G81 |
| G28 | G71 | G89 |
| G29 | G73 | G102 |
| G30 | G80 | G105 |
| G31 | G83 | G113 |
| G32 | G86 | G117 |
| G44 | G87 | G131 |
| G51 | G88 | G164 |
| G56 | G92 |  |
| G63 | G93 |  |
| G64 | G96 |  |
| G72 | G100 |  |
| G74 | G103 |  |
| G75 | G108 |  |
| G78 | G110 |  |
| G79 | G112 |  |
| G82 | G114 |  |
| G84 | G118 |  |
| G85 | G119 |  |
| G90 | G124 |  |
| G91 | G130 |  |
| G94 | G139 |  |
| G95 | G141 |  |
| G97 | G142 |  |
| G98 | G146 |  |
| G101 | G149 |  |
| G104 |  |  |
| G106 |  |  |
| G107 |  |  |
| G109 |  |  |
| G111 |  |  |
| G115 |  |  |
| G116 |  |  |
| G120 |  |  |
| G121 |  |  |
| G122 |  |  |
| G123 |  |  |
| G125 |  |  |
| G126 |  |  |
| G127 |  |  |
| G128 |  |  |
| G129 |  |  |
| G132 |  |  |
| G133 |  |  |
| G134 |  |  |
| G135 |  |  |
| G136 |  |  |
| G137 |  |  |
| G138 |  |  |
| G143 |  |  |
| G144 |  |  |
| G145 |  |  |
| G147 |  |  |
| G148 |  |  |
| G150 |  |  |
| G161 |  |  |
| G162 |  |  |
| G163 |  |  |
| G165 |  |  |
| G166 |  |  |
| G167 |  |  |
| G168 |  |  |
| G169 |  |  |
| G170 |  |  |
