# Supplementary material for: Association analysis between agronomic traits and AFLP markers in a wide germplasm of proso millet (Panicum miliaceum L.) under normal and salinity stress conditions
Source: BMC Plant Biol. 2020 Sep 15;20:427. doi: 10.1186/s12870-020-02639-2 (PMC7493190; doi:10.1186/s12870-020-02639-2)
Supplement: Supplementary file 4 — Additional file 4: Table S4. Association analysis of 143 proso millet genotypes under normal and salt stress conditions based on the MLM model. [file 12870_2020_2639_MOESM4_ESM.docx]

| **Additional file 4: Table S4**. Association analysis of 143 proso millet genotypes under normal and salt stress conditions based on the MLM model**.** | | | | | | | |
| --- | --- | --- | --- | --- | --- | --- | --- |
| **Normal conditions** | | | | **Salt stress conditions** | | | |
| **Trait** | **Marker** | **P** | **R^2^ (%)** | **Trait** | **Marker** | **P** | **R^2^ (%)** |
|  |  |  |  |  |  |  |  |
| Seed yield | M14/E10-60 | 0.0052 | 40.58 | Seed yield | M14/E10-64 | 0.0035 | 37.37 |
|  | M14/E10-45 | 0.0059 | 40.47 |  | M4/E11- 4 | 0.0056 | 36.94 |
|  | M4/E11-71 | 0.0072 | 40.3 |  | M3/E36- 2 | 0.0084 | 36.57 |
|  | M4/E11-70 | 0.0085 | 40.15 |  |  | | |
|  | M59/E36-1 | 0.0091 | 40.09 |  |  |  |  |
|  |  |  |  |  |  |  |  |
| Forage yield | M14/E10-45 | 0.0038 | 40.83 | Forage yield | M14/E10-64 | 0.0044 | 38.17 |
|  | M14/E10-60 | 0.0055 | 40.52 |  | M3/E36- 32 | 0.0051 | 38.03 |
|  | M4/E11-44 | 0.0069 | 40.31 |  | M14/E10-78 | 0.0082 | 37.6 |
|  | M4/E11-71 | 0.008 | 40.18 |  |  |  |  |
|  | M59/E11-70 | 0.0084 | 40.14 |  |  |  |  |
|  | M59/E11-75 | 0.0084 | 40.13 |  |  |  |  |
| Number of leaves per plant | M3/E10- 3 | 0.0017 | 40.99 | Number of leaves per plant | M4/E10-8 | 0.0031 | 41.46 |
|  | M59/E36- 3 | 0.0046 | 40.1 |  | M59/E36- 3 | 0.0037 | 41.31 |
|  | M59/E11-47 | 0.005 | 40.03 |  | M59/E36-71 | 0.0037 | 41.3 |
|  | M14/E10-69 | 0.0069 | 39.74 |  | M3/E10-3 | 0.0039 | 41.25 |
|  | M14/E10-40 | 0.007 | 39.73 |  | M59/E36-74 | 0.0052 | 41.01 |
|  |  |  |  |  | M14/E10-27 | 0.0057 | 40.92 |
|  |  |  |  |  | M14/E10-40 | 0.0077 | 40.66 |
| Plant height | M4/E11- 44 | 0.0024 | 41.06 | Plant height | M59/E10-23 | 0.0047 | 38.78 |
|  | M59/E36-60 | 0.0043 | 40.55 |  | M59/E11-45 | 0.0048 | 38.75 |
|  | M4/E36-4 | 0.0047 | 40.46 |  | M59/E36-14 | 0.0093 | 38.15 |
|  | M59/E36-76 | 0.0064 | 40.18 |  |  |  |  |
| Seed germination percentage | M4/E36- 67 | 0.0032 | 39.2 | Seed germination percentage | M3/E10- 2 | 0.0018 | 41.16 |
|  | M14/E10-67 | 0.0034 | 39.16 |  | M4/E11- 1 | 0.0026 | 40.84 |
|  | M4/E11-78 | 0.0047 | 38.85 |  | M4/E36- 2 | 0.0032 | 40.65 |
|  | M14/E10-44 | 0.0058 | 38.67 |  | M14/E11-44 | 0.0068 | 39.99 |
|  | M59/E10-64 | 0.0092 | 38.24 |  | M4/E36-45 | 0.0016 | 39.98 |
|  |  |  |  |  | M4/E10-79 | 0.0084 | 39.8 |
| Flag leaf length | M59/E36-39 | 0.0099 | 38.62 | Flag leaf length | 39.78 | 39.78 | 39.78 |
|  |  | | |  | M59/E11-55 | 0.0065 | 38.71 |
|  |  |  |  |  | M59/E10-44 | 0.0074 | 38.47 |
|  |  |  |  |  | M59/E10-24 | 0.0097 | 38.36 |
|  |  |  |  |  | M4/E11-11 | 0.0016 | 38.11 |
| Number of panicle branches | M4/E11-44 | 0.002 | 37.39 | Number of panicle branches | M4/E36-45 | 0.0017 | 38.71 |
|  | M59/E36-37 | 0.0039 | 36.73 |  | M3/E10-71 | 0.0025 | 38.36 |
|  | M3/E36-17 | 0.0077 | 36.1 |  | M4/E11-76 | 0.0027 | 38.31 |
|  | M4/E11-40 | 0.0082 | 36.04 |  | M4/E11-83 | 0.0044 | 37.83 |
|  | M3/E11-13 | 0.0092 | 35.92 |  | M59/E11-45 | 0.0066 | 37.47 |
|  | M4/E11-61 | 0.0093 | 35.92 |  | M3/E36-41 | 0.007 | 37.41 |
| Flag leaf width | M59/E10-83 | 0.0071 | 42.73 | Flag leaf width | M4/E11-15 | 0.0019 | 42.8 |
|  | M59/E11-82 | 0.0094 | 42.49 |  | M4/E11-25 | 0.006 | 41.83 |
|  |  |  |  |  | M14/E10-2 | 0.0095 | 41.43 |
| Number of tiller | M59/E10-83 | 0.00087439 | 42.95 | Number of tiller | M4/E11-25 | 0.0046 | 41.57 |
|  | M14/E10-31 | 0.0043 | 41.55 |  | M4/E11-15 | 0.0064 | 41.28 |
|  | M59/E36-48 | 0.0075 | 41.07 |  | M59/E36-48 | 0.0092 | 40.97 |
| panicle length | M4/E10- 25 | 0.0031 | 40.6 | panicle length | M4/E10- 8 | 0.0033 | 40.37 |
|  | M59/E11-15 | 0.0043 | 40.3 |  | M4/E10- 11 | 0.0061 | 39.83 |
|  | M4/E10-48 | 0.006 | 40 |  | M59/E11-18 | 0.0067 | 39.73 |
|  | M59/E36-81 | 0.0089 | 39.65 |  | M59/E36-31 | 0.0086 | 39.51 |
| Number of plant on the line | M4/E36- 67 | 0.0019 | 40.87 | Number of plant on the line | M14/E11-67 | 0.0019 | 40.46 |
|  | M4/E11- 45 | 0.0051 | 40 |  | M3/E36- 41 | 0.0036 | 39.86 |
|  | M59/E11-9 | 0.006 | 39.86 |  | M4/E11-31 | 0.0093 | 39.01 |
|  | M14/E10-19 | 0.0088 | 39.51 |  | M59/E11-39 | 0.0098 | 38.96 |
| Main panicle seed weight | M59/E10-22 | 0.0025 | 39.91 | Main panicle seed weight | M59/E10-22 | 0.0031 | 39.36 |
|  | M59/E11-31 | 0.0039 | 39.51 |  | M3/E36-45 | 0.0055 | 38.85 |
|  | M4/E11-71 | 0.0048 | 39.33 |  | M4/E11-71 | 0.0061 | 38.75 |
|  | M3/E36-45 | 0.0048 | 39.32 |  | M59/E11-31 | 0.0066 | 38.68 |
|  | M4/E11-61 | 0.0084 | 38.82 |  | M4/E11-61 | 0.0071 | 38.62 |
|  |  |  |  |  | M4/E11-72 | 0.0084 | 38.46 |
| 1000-seed weight | M3/E36-45 | 0.0027 | 38.78 | 1000-seed weight | M3/E36-41 | 0.0015 | 39.64 |
|  | M14/E11-27 | 0.0046 | 38.3 |  | M14/E11-44 | 0.0029 | 39.04 |
|  | M14/E11-44 | 0.0058 | 38.08 |  | M14/E11-27 | 0.0052 | 38.5 |
|  | M14/E10-69 | 0.0095 | 37.63 |  | M59/E11-54 | 0.0055 | 38.46 |
|  |  |  |  |  | M14/E10-69 | 0.0091 | 38 |
| Harvest index | M4/E11-44 | 0.002 | 43.71 | Harvest index | M14/E11-23 | 0.0043 | 43.3 |
|  | M4/E11-79 | 0.0032 | 43.28 |  | M3/E36-41 | 0.006 | 43.01 |
|  | M3/E10-31 | 0.0045 | 43 |  | M4/E36-44 | 0.0068 | 42.91 |
|  | M4/E36-31 | 0.0046 | 42.98 |  | M4/E10-8 | 0.0071 | 42.87 |
|  | M14/E10-3 | 0.0058 | 42.79 |  | M3/E11-13 | 0.0084 | 42.73 |
|  | M3/E10-2 | 0.0081 | 42.51 |  |  | | |
|  | M14/E11-23 | 0.0088 | 42.44 |  |  |  |  |
| Biological yield | M14/E10-45 | 0.0039 | 40.88 | Biological yield | M14/E10-64 | 0.0031 | 37.72 |
|  | M14/E10-60 | 0.0055 | 40.56 |  | M3/E36-2 | 0.0052 | 37.24 |
|  | M4/E11-44 | 0.0073 | 40.32 |  |  | | |
|  | M4/E11-71 | 0.0079 | 40.24 |  |  |  |  |
|  | M59/E11-70 | 0.0085 | 40.18 |  |  |  |  |
|  | M59/E11-75 | 0.0087 | 40.15 |  |  |  |  |
