# Supplementary material for: Association analysis between agronomic traits and AFLP markers in a wide germplasm of proso millet (Panicum miliaceum L.) under normal and salinity stress conditions
Source: BMC Plant Biol. 2020 Sep 15;20:427. doi: 10.1186/s12870-020-02639-2 (PMC7493190; doi:10.1186/s12870-020-02639-2)
Supplement: Supplementary file 5 — Additional file 5: Table S5. Geographical location and code of the collected proso millet (Panicum miliaceum L.) genotypes. [file 12870_2020_2639_MOESM5_ESM.docx]

| **Additional file 5: Table S5.** Geographical location and code of the collected proso millet (*Panicum miliaceum* L.) genotypes. | | | | | |
| --- | --- | --- | --- | --- | --- |
| **Code of genotypes** | **Location** | **Code of genotypes** | **Location** | **Code of genotypes** | **Location** |
| G1 | Yazd 2 | G49 | Sari 4 | G97 | Yazd 4 |
| G2 | Tabas 3 | G50 | Shiraz 1 | G98 | Khuzestan 2 |
| G3 | Tabas 4 | G51 | Shiraz 3 | G99 | Khorramabad 1 |
| G4 | Gorgan 1 | G52 | Shiraz 4 | G100 | Ahvaz 2 |
| G5 | Tabriz | G53 | Shiraz 7 | G101 | Ahvaz 3 |
| G6 | Shiraz 2 | G54 | Ilam 1 | G102 | Ahvaz 4 |
| G7 | Ilam 3 | G55 | Ilam 2 | G103 | Sanandaj 1 |
| G8 | Gilan 5 | G56 | Gorgan 2 | G104 | Sanandaj 2 |
| G9 | Zabol3 | G57 | Ilam 4 | G105 | Khorramabad 2 |
| G10 | Yazd 1 | G58 | Zanjan | G106 | Khuzestan 3 |
| G11 | Kerman 2 | G59 | Marvdasht | G107 | Sanandaj 3 |
| G12 | Ferdows | G60 | Gilan 1 | G108 | Khuzestan 4 |
| G13 | Quchan | G61 | Saravan | G109 | Sanandaj 4 |
| G14 | Birjand 1 | G62 | Gilan 4 | G110 | Khuzestan 5 |
| G15 | Birjand 3 | G63 | Chabahar 2 | G111 | Kurdistan 1 |
| G16 | Birjand 2 | G64 | Zahedan 2 | G112 | Kurdistan 2 |
| G17 | Birjand 4 | G65 | Gilan 2 | G113 | Kurdistan 3 |
| G18 | Tabas 1 | G66 | Urmia 2 | G114 | Khuzestan 6 |
| G19 | Tabas 2 | G67 | Isfahan 2 | G115 | Kurdistan 4 |
| G20 | Shahrekord 6 | G68 | Gilan 3 | G116 | Khuzestan 7 |
| G21 | Shahrekord 1 | G69 | Qazvin 1 | G117 | Bojnurd |
| G22 | Shahrekord 2 | G70 | Borujerd | G118 | Gonbad Kavus |
| G23 | Shahrekord 3 | G71 | Zabol2 | G119 | Kurdistan 5 |
| G24 | Mashhad 5 | G72 | Isfahan 3 | G120 | Ardabil 3 |
| G25 | Shahrekord 4 | G73 | Zabol4 | G121 | Khuzestan 8 |
| G26 | Isfahan 1 | G74 | Arak 1 | G122 | Ardabil 4 |
| G27 | Naein | G75 | Ahvaz 1 | G123 | Qazvin 2 |
| G28 | Shahrekord 5 | G76 | Arak 2 | G124 | Qazvin 3 |
| G29 | Darab | G77 | Hamedan 1 | G125 | Qazvin 4 |
| G30 | Mashhad 6 | G78 | Sari 5 | G126 | Kurdistan 6 |
| G31 | Chabahar 1 | G79 | Mazandaran 2 | G127 | Kurdistan 7 |
| G32 | Mashhad 1 | G80 | Mazandaran 3 | G128 | Kurdistan 8 |
| G33 | Mashhad 2 | G81 | Mazandaran 5 | G129 | Ardabil 5 |
| G34 | Mashhad 3 | G82 | Mazandaran 4 | G130 | Kerman 5 |
| G35 | Mashhad 4 | G83 | Mazandaran 1 | G131 | Kerman 1 |
| G36 | Tabriz | G84 | Hamedan 2 | G132 | Mashhad 7 |
| G37 | Sarbaz | G85 | Hamedan 3 | G133 | Mashhad 10 |
| G38 | Tabriz | G86 | Hamedan 4 | G134 | Shiraz 6 |
| G39 | Jahrom | G87 | Zahedan 3 | G135 | Pishahang |
| G40 | Tabriz | G88 | Yazd 3 | G136 | Kahnooj |
| G41 | Tabriz | G89 | Kerman 3 | G137 | Shahrbabak |
| G42 | Ardabil 1 | G90 | Kerman 4 | G138 | Zahedan 1 |
| G43 | Ardabil 2 | G91 | Meybod | G139 | Zabol1 |
| G44 | Yasuj | G92 | Shiraz 5 | G140 | Rābor |
| G45 | Urmia 1 | G93 | Bandar Anzali | G141 | Golbāf |
| G46 | Sari 1 | G94 | Khuzestan 1 | G142 | Sixty days period |
| G47 | Sari 2 | G95 | Neyshabur | G143 | Ninety days period |
| G48 | Sari 3 | G96 | Ardakan |  |  |
